# Supplementary material for: Serum FSH as a Useful Marker for the Differential Diagnosis of Ovarian Granulosa Cell Tumors
Source: Cancers (Basel). 2022 Sep 15;14(18):4480. doi: 10.3390/cancers14184480 (PMC9496984; doi:10.3390/cancers14184480)
Supplement: Supplementary file 1 [file cancers-14-04480-s001.zip › cancers-1880175-supplementary.pdf]

Supplemental Table S1. Histolgic subtypes.

**Histologic subtype (n=471)**		**n**
Serous tumors		
	Cystadenoma	10
	Borderline tumor	7
	Carcinoma	116
Mucinous tumors		
	Cystadenoma	40
	Borderline tumor	29
	Carcinoma	19
Endometrioid tumors		
	Adenofibroma	3
	Borderline tumor	2
	Carcinoma	49
Clear cell tumors		
	Carcinoma	73
Seromucinous tumors		
	Adenofibroma	1
	Borderline tumor	8
	Carcinoma	4
Brenner tumors		
	Benign	4
	Malignant	2
Other carcinomas		
	Undifferentiated carcinoma	1
	Carcinosarcoma	10
Mesenchymal tumors		
	Endometrioid stromal sarcoma, low grade	1
Mixed epithelial and mesenchymal tumors		
	Adenosarcoma	1
Sex cord-stromal tumors		
Pure stromal tumors		
	Fibroma	19
	Thecoma	4
	Leydig cell tumor	1
Pure sex cord tumors		
	Adult granulosa cell tumor	13
Germ cell tumors		
	Teratoma, benign	18
	Immature teratoma	1
	Dysgerminoma	1
	Yolk sac tumor	1
	Struma ovarii	1
	Strumal carcinoid	1
Miscellaneous tumors		
	Small cell carcinoma	1
Endometriosis and derived tumors		
	Endometriosis	30

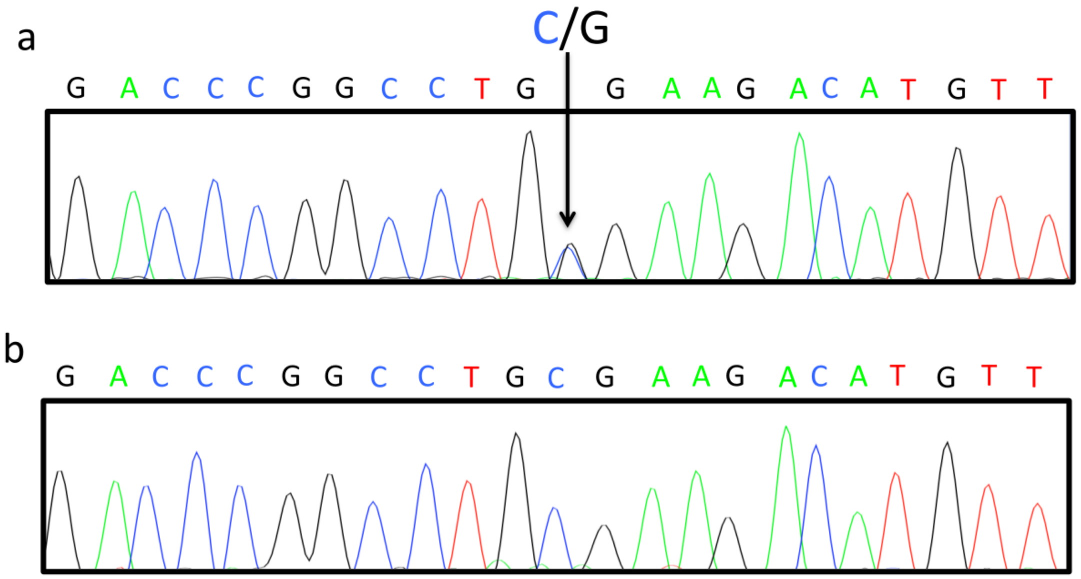

Supplementary Figure S1. Mutations of FOXL2 gene codon 134 (p.C134W) in granulosa cell tumors (GCT). Direct DNA sequencing analyses of adult-type GCT with the (a) FOXL2 mutation and (b) without FOXL2 mutation.

Supplemental Table S2. Serum hormone levels of GCT group and non GCT group.

Characteristic	GCT group (n=13)		P value	non-GCT group (n=458)		P value
	Premenopausal (n = 7)	Postmenopausal (n = 6)		Premenopausal (n = 155)	Postmenopausal (n = 303)	
LH (IU/L)	5.0 [0.3-33]	3.1 [0.1-24]	0.47	4.1 [0.1-49]	19 [0.1-70]	< .001
FSH (IU/L)	0.9 [0.1-1.8]	0.4 [0.1-2.0]	0.94	6.0 [0.1-89]	48 [2.2-150]	< .001
Teststerone (nmol/L)	0.8 [0.5-3.9]	0.5 [0.3-0.9]	0.19	0.3 [0.1-10]	0.3 [0.1-11]	0.92
Estradiol (pmol/L)	83 [17-370]	81 [52-280]	0.81	56 [10-650]	16 [10-120]	< .001
Progesterone (pmol/L)	0.5 [0.3–4.4]	0.4 [0.1-0.7]	0.25	0.3 [0.1–18]	0.2 [0.1-4.7]	< .001

Values are presented as median [range].

GCT, granulosa cell tumor; LH, luteinizing hormone; FSH, follicle stimulating hormone
